# Awareness of caregivers of geriatric deficits among older people—the results of a cross-sectional study in Krakow, Poland

**DOI:** 10.1186/s12875-022-01801-z

**Published:** 2022-07-26

**Authors:** Anna Pachołek, Karolina Piotrowicz, Jerzy Gąsowski, Tomasz Tomasik

**Affiliations:** 1 Department of Family Medicine, Jagiellonian University Medical College, Bocheńska 4 Street, 31-061 Krakow, Poland; 2 The College of Family Physicians in Poland, Warszawa, Poland; 3 Department of Internal Medicine and Gerontology, Jagiellonian University Medical College, Krakow, Poland

**Keywords:** General practitioner, Primary care, Caregivers, Comprehensive geriatric assessment, Poland

## Abstract

**Background:**

It seems that caregivers (CGs) may be a reliable source of information for determining health condition of seniors. This might be important for general practitioners (GPs) and facilitate them conducting comprehensive geriatric assessment (CGA). The objectives of our study were to: compare populations of older patients with and without CGs, characterise the group of CGs, establish whether CGs are aware of patients’ deficiencies in areas of CGA.

**Methods:**

Patients aged at least 65 years underwent CGA using eight tools in GPs’ practices in and around Krakow, Poland. Seniors were divided into two groups: with and without CGs. CGs filled in an authors’ questionnaire on their data and assessed seniors in eight domains corresponding to the tests used in CGA. Patients with and without CGs were also compared in terms of CGA results and basic demographic and medical data. Subjective CGs’ responses were compared with objective CGA results.

**Results:**

We conducted CGA on 438 senior patients. Two hundred fifty eight (59%) of them were classified as patients with CGs. Patients with CGs were older, less educated, more often lived in rural areas and were more frequently in a relationship (as all *p* < 0.05). In seniors with CGs, the results of frailty (*p* < 0.008) and insomnia scales (*p* = 0.049) were significantly worse. Mostly, CGs could properly assess seniors in basic and complex living activities and nutritional status. They were less precise in determining deficits like depressive tendency and insomnia.

**Conclusions:**

CGs’ assessment of older patients can be a valuable source of information about seniors and can be helpful in diagnosing important health issues. CGs have difficulties when asked to properly assess depression and insomnia in the older adults they care for and their answers do not always correspond with the results of CGA. GPs should pay more attention to the needs of CGs themselves and provide them with the necessary knowledge about caring for older people.

**Supplementary Information:**

The online version contains supplementary material available at 10.1186/s12875-022-01801-z.

## Background

An ageing of society and longer life expectancy have resulted in a rapidly increasing number of older people in Poland [1, 2]. A lack of sufficient number of geriatricians and general practitioners (GPs) impedes the holistic assessment of seniors and physicians often have to rely on the informational help of patients’ families [3].

Therefore, the interest of researchers is beginning to focus on caregivers (CGs), who can serve as a trustworthy and valued source of information contributing to patient assessment. The usefulness of deriving health-related data concerning older people from their CGs is a novel field for investigation [4, 5].

Health care professionals (HPs) use comprehensive geriatric assessment (CGA) to recognize the physical, mental, emotional and socioeconomic needs of older people. This complex tool evaluates seniors’ capabilities and limitations and should lead to the development of a long-term plan for patients’ treatment and rehabilitation [6]. Some areas of such assessment obtained from a CG may be useful in busy clinical practice [7–9].

## Aims

The goals of our study were as follows: comparison of patients with and without CGs in terms of demographic and medical issues; characterisation of the group of CGs caring for senior patients; establishing whether CGs are aware of patients’ deficiencies in the areas of CGA.

## Methods

### Design and setting

A cross-sectional questionnaire study was conducted in Krakow, the second-largest city in Poland, and nearby villages, among patients aged at least 65 years and their CGs.

Participants were recruited in 15 primary care practices. These were randomly selected from all practices cooperating with the Jagiellonian University Medical College (*n* = 47). The practices were similar to each other. They were private GP practices who were under contract with the National Health Fund for the provision of primary health care services. The largest of the practices delivered medical care for approximately 5,700 patients, and the smallest for about 2,300 people. All institutions provided general care for patients, regardless of their age. The median number of patients per practice was 3439 (2580–4724).

The data were collected in the period from April 2018 to April 2019.

### Population studied

Senior patients and their CGs were recruited into the study. A consecutive sample of 30 patients attending general practices was selected. Patients were eligible to participate if they were: at least 65 years old, on the GPs’ patient list, able to come to the practice unaided, spoke Polish and gave signed informed consent. Four practices did not manage to recruit a total of 12 patients by the date set as the end of data collection, hence the required 450 patients were not obtained.

Each patient was asked to indicate their CG, if they had one. Based on this parameter, seniors were divided into two groups, those with and those without CGs. A CG was defined as a relative or helper (professional or otherwise) indicated by the patient who helps if help is needed and is knowledgeable about the patient’s health and social circumstances.

### Research tools and procedure

The study consisted of two parts. The first was the patient’s CGA conducted by the researcher (a trained physician). During the assessment, eight tools were used, these being: the Activities of Daily Living (ADL) and Instrumental Activities of Daily Living (IADL) which evaluate independence in basic and more complex every day activities [10, 11]; Mini-Mental State Examination (MMSE), assessing cognitive impairment [12]; Geriatric Depression Scale (GDS) which is a useful screening tool to facilitate assessment of depression in older adults [13]; Timed Up and Go Test (TT), evaluating mobility and risk of falls [14]; Mini Nutritional Assessment Short Form (MNA), estimating nutritional status [15]; Clinical Frailty Scale (CFS) which assesses the risk of frailty syndrome [16] and Athens Insomnia Scale (AIS) which assesses sleeplessness [17].

In addition to CGA, a questionnaire developed by the authors was used to collect patients’ data. It consisted of 20 questions and gathered biometric as well as medical information about seniors. Variables like: gender, weight, height, place of residence, education, physical activity, smoking, chronic diseases, medication intake, use of medical services etc. were collected. More detailed descriptions of questionnaires used in this study are shown in our previous article [18].

The second part of the study concerned patients’ CGs. A questionnaire developed by the authors (two GPs and one geriatrician) was tested in a group of 10 CGs in one practice and assessed as understandable. Its validity was assessed qualitatively based on the opinions of the CGs and researchers. Ten CGs provided feedback on the questionnaire’s clarity, length and wordiness and this confirmed high face validity. Content validity was tested by the research team. The content of our questionnaire was compared with that of other tools for CGs of patients used in Poland [19, 20]. Our instrument was similar to these tools and it did not contain any deficits in important issues.

The questionnaire consisted of 2 open and 19 closed questions [Additional file]. It collected basic information about the participant. In the next part of the form, the questions were devoted to the possible existence of problems in eight areas corresponding to CGA. These aspects were as follows: level of independence in the area of basic activities and instrumental activities of daily living, cognitive functions, depressive disorders, mobility, nutritional status, features of frailty syndrome and sleeplessness. The answers to these closed questions were limited to three possibilities: “Yes”, “No” and “I can’t assess”. The last part of the form contained questions whether CGs used assistance in caring for the senior, if any of the HPs had spoken to them about how to care for older people and if they would like to receive such information.

CGs answers regarding CGA were classified as correct and incorrect. If the subjective CG response matched the objective CGA results, it was categorised as the correct assessment. For example, if the ADL questionnaire revealed deficits and the CG’s answer to the question: "Is the patient capable of independent living?" was "No", it was qualified as a correct answer. If the answer to the same question was "Yes" or "I can’t assess" it would be qualified as incorrect. Other CGs’ answers were dichotomised in similar way (one group with a positive answer to the question, the second with a negative answer to the question or answer “I can’t assess”).

Patients with CGs and without CGs were also compared in terms of CGA results and basic demographic and medical data.

### Statistical analysis

Statistical analysis was performed using R, version 4.0.3 [21]. The Shapiro–Wilk test was used as a normality measure. Comparison of the values of qualitative variables in the groups was performed using the chi-square test (with Yates’s correction for 2 × 2 tables) or Fisher’s exact test where low expected frequencies appeared. Comparison of the values of quantitative variables in the two groups was performed using the Mann–Whitney test. For each of the dichotomous variables (concordance between CGs and patients in assessment of various capabilities) two separate univariate logistic regressions were conducted—one with CGs’ age and another with CGs’ gender, both as independent variables. Two separate univariate logistic regressions (one for CGs’ age and one for CGs’ gender) were additionally conducted for CGs’ traits. The results are presented in the form of OR parameter values with a 95% confidence interval. Statistical significance was set at 0.05.

## Results

We conducted CGA on 438 senior patients. Two hundred fifty eight (59%) of them were classified as patients with CGs and 180 (41%) as patients without CGs.

### Characteristics of patients with and without CGs

Detailed characteristics of patients with and without CGs and a comparison of their results of eight CGA tests are shown in Table 1.

**Table 1:** Comparison of patients with and without caregivers in terms of sociodemographic and medical parameters and CGA results

Parameter	Patients		*p*
	**With caregivers (*N* = 258)**	**Without caregivers (*N* = 180)**	
Age [years]			
mean ± SD	76.75 ± 8.28	74.04 ± 6.93	*p* = 0.001 *
median	76	72.5	
quartiles	69—84	68—79	
Gender			
Female	165 (63.95%)	111 (61.67%)	*p* = 0.699
Male	93 (36.05%)	69 (38.33%)	
Residence			
Rural area	103 (39.92%)	39 (21.67%)	*p* < 0.001 *
Urban area	155 (60.08%)	141 (78.33%)	
BMI [kg/m2]			
Underweight	5 (1.94%)	0 (0.00%)	*p* = 0.058
Normal weight	61 (23.64%)	59 (32.78%)	
Overweight	117 (45.35%)	75 (41.67%)	
Obesity	75 (29.07%)	46 (25.56%)	
Education			
Primary	71 (27.52%)	19 (10.56%)	*p* < 0.001 *
Secondary	86 (33.33%)	72 (40.00%)	
Vocational	48 (18.60%)	29 (16.11%)	
University (incomplete)	4 (1.55%)	8 (4.44%)	
University	49 (18.99%)	52 (28.89%)	
Marital status			
Single	107 (41.47%)	98 (54.44%)	*p* = 0.01 *
In relationship	151 (58.53%)	82 (45.56%)	
Multiple medication [more than 5 medicaments per day]			
No	134 (51.94%)	80 (44.44%)	*p* = 0.148
Yes	124 (48.06%)	100 (55.56%)	
ADL [points]			
mean ± SD	5.53 ± 1.24	5.95 ± 0.24	*p* < 0.001 *
median	6	6	
quartiles	6—6	6—6	
IADL [points]			
mean ± SD	20.55 ± 4.73	23.07 ± 1.69	*p* < 0.001 *
median	23	24	
quartiles	18—24	23—24	
MMSE [points]			
mean ± SD	25.6 ± 5.04	26.99 ± 2.84	*p* = 0.103
median	27	28	
quartiles	24—29	26—29	
GDS [points]			
mean ± SD	4.64 ± 3.38	4.06 ± 3.14	*p* = 0.062
median	4	3	
quartiles	2—7	2—5	
TT [s]			
mean ± SD	14.7 ± 10.42	12.58 ± 5.32	*p* = 0.288
median	11.5	11	
quartiles	9—16	9—15	
MNA [points]			
mean ± SD	12.27 ± 2.21	12.44 ± 1.94	*p* = 0.755
median	13	13	
quartiles	11.25—14	12—14	
CFS [points]			
mean ± SD	3.28 ± 1.57	2.78 ± 1.14	*p* = 0.008 *
median	3	3	
quartiles	2—4	2—4	
AIS [points]			
mean ± SD	5.91 ± 4	5.49 ± 4.45	*p* = 0.049 *
median	5	4	
quartiles	3—8	2—8	

p—Mann–Whitney test for quantitative variables, chi-squared or Fisher’s exact test for qualitative variables

^*^Statistically significant (*p* < 0.05)

Generally, patients with CGs were older, less educated, more often lived in rural areas and were more frequently in a relationship. All these differences were statistically significant. There were no statistically significant relationships between having a CG and patients’ gender, BMI and multiple medication. In seniors with CGs, the results of CFS and AIS were significantly higher and the results of ADL and IADL were significantly lower.

### CGs’ characteristics

The mean age of CGs was 59.37 ± 0.5 years. Most of them were women (182; 70%). One hundred and forty one of them (55%) were below 65 years old, while 117 (45%) were over 64. Almost two thirds of CGs (167; 65%) lived with the examined seniors. Detailed characteristics of CGs are described in Table 2.

**Table 2:** Characteristics of the caregivers

Parameter	Gender		*p*
	**Female (*N* = 182)**	**Male (*N* = 76)**	
Age [yrs]			
mean ± SD	58.36 ± 14.21	61.78 ± 15.02	*p* = 0.044 *
median	60	68	
quartiles	48—68.75	47.5—72	
What is your relationship with the person you care for?			
Spouse/partner	66 (36.26%)	41 (53.95%)	*p* = 0.021 *
Sibling	6 (3.30%)	2 (2.63%)	
Child	68 (37.36%)	23 (30.26%)	
Daughter-in-law/Son-in-law	15 (8.24%)	0 (0.00%)	
Acquaintance/friend/neighbour	10 (5.49%)	5 (6.58%)	
Other	17 (9.34%)	5 (6.58%)	
Living with senior			
No	67 (36.81%)	24 (31.58%)	*p* = 0.51
Yes	115 (63.19%)	52 (68.42%)	
Using help from others			
No	150 (82.42%)	68 (89.47%)	*p* = 0.215
Yes	32 (17.58%)	8 (10.53%)	
Willingness to receive help from others			
No	149 (81.87%)	65 (85.53%)	*p* = 0.596
Yes	33 (18.13%)	11 (14.47%)	
Decrease in quality of life			
No	150 (82.42%)	67 (88.16%)	*p* = 0.336
Yes	32 (17.58%)	9 (11.84%)	
Consultations with HP			
No	132 (72.53%)	67 (88.16%)	*p* = 0.01 *
Yes	50 (27.47%)	9 (11.84%)	
Willingness to receive more information			
No	95 (52.20%)	49 (64.47%)	*p* = 0.094
Yes	87 (47.80%)	27 (35.53%)	

p—Mann–Whitney test for quantitative variables, chi-squared or Fisher’s exact test for qualitative variables

^*^ Statistically significant (*p* < 0.05)

Logistic regression models showed that CG’s age was a significant predictor of the chance of cohabitation with the patient (OR = 1.071, 95% CI:1.049 -1.095). Age (OR = 0.971, 95% CI: 0.952–0.991) and gender (OR = 0.355, 95% CI: 0.164—0.765) were significant predictors of the likelihood for consulting HPs. There were no statistically significant relationships between the gender of CGs and use of help from others, willingness to receive help from others, decrease in CGs’ quality of life and willingness to receive more information from HP about taking care of seniors. Also, no relationship was found between the age of CGs and any of the previously listed possibilities (in all cases *p* > 0.05).

### CGs’ assessment of the seniors in eight areas of CGA

**Fig. 1:**
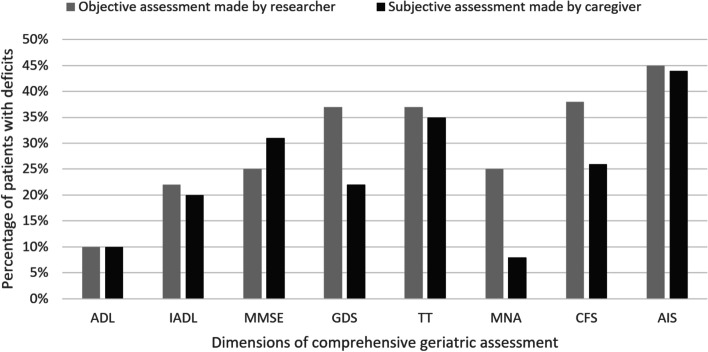
The percentage of patients with deficits in eight dimensions of comprehensive geriatric assessment in objective assessment made by the researcher and subjective assessment made by the caregiver. ADL—the Activities of Daily Living, IADL—the Instrumental Activities of Daily Living, MMSE—Mini-Mental State Examination, GDS—Geriatric Depression Scale, TT—Timed Up and Go Test, MNA—Mini Nutritional Assessment Short Form, CFS—Clinical Frailty Scale, AIS—Athens Insomnia Scale

A comparison of patient deficits in eight CGA domains in objective CGA performed by the researcher and subjective assessment by the CG is presented on Fig. 1. CGs most often indicated deficits in terms of insomnia and patients’ mobility. Similarly, a high percentage of deficits in those domains were revealed in objective CGA; however, CGs less frequently drew attention to seniors’ problems with malnutrition and depression.

**Fig. 2:**
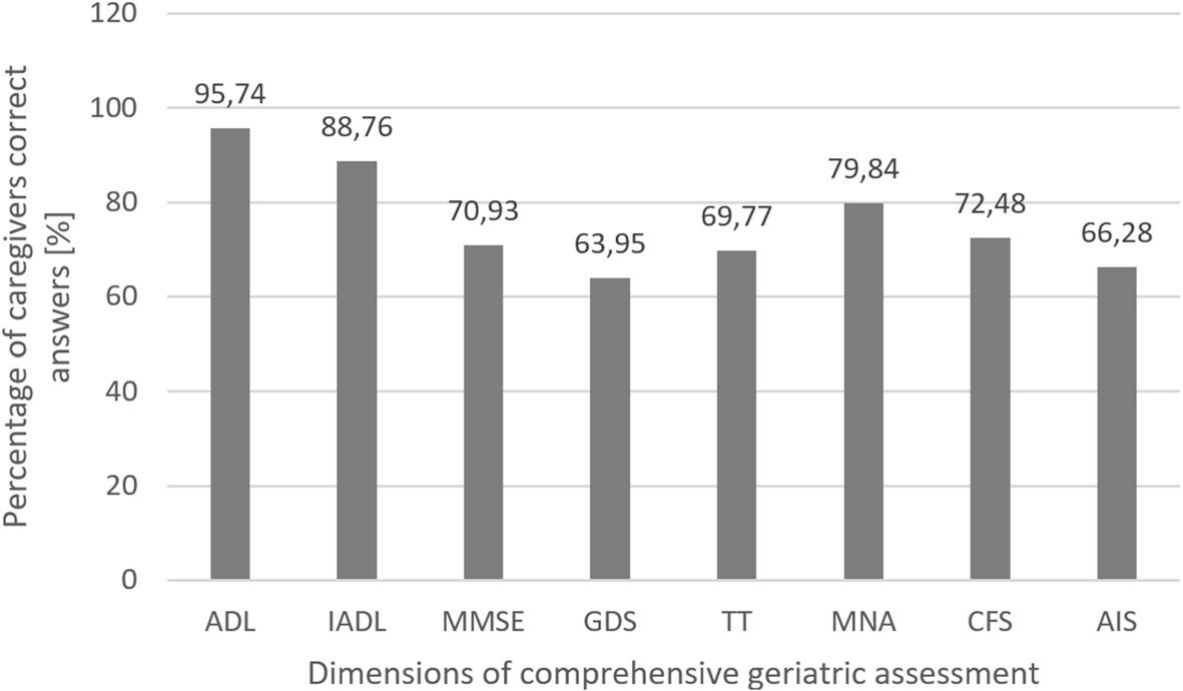
The percentage of correct caregivers’ assessment in eight dimensions of comprehensive geriatric assessment. ADL—the Activities of Daily Living, IADL—the Instrumental Activities of Daily Living, MMSE—Mini-Mental State Examination, GDS—Geriatric Depression Scale, TT—Timed Up and Go Test, MNA—Mini Nutritional Assessment Short Form, CFS—Clinical Frailty Scale, AIS—Athens Insomnia Scale

The correctness of CGs’ subjective assessment of deficiencies in seniors is shown in Fig. 2. In most cases, CGs could properly assess seniors in basic and complex living activities and nutritional status. On the other hand, they were less precise in determining deficits in more psychological aspects like depressive tendency and insomnia.

Logistic regression models showed that neither CGs’ age nor gender were important predictors of the chance of correct assessment in ADL, IADL, MNA, AIS, CFS, TT and MMSE (in all cases *p* > 0.05) Table 3. Only in the case of GDS, did it emerge that CGs’ age was important in recognising depressive tendency, so each annual increase in the age of the CG increased this chance by 2.3% (OR = 1.023, 95% CI:1.005–1.041).

**Table 3:** Caregivers’ gender and age as predictors of the chance of correct assessment in dimensions of CGA

Parameter	OR (95%CI), *p* for caregivers’ gender and age	
	**Males vs Females**	**Age [yrs]**
Basic activities measured by ADL	1.119 (0.289–4.336), *p* = 0.871	0.988 (0.947–1.031), *p* = 0.582
Complex living skills measured by IADL	2.157 (0.791–5.883), *p* = 0.133	1.004 (0.978–1.031), *p* = 0.778
Cognitive functions measured by MMSE	1.333 (0.727–2.447), *p* = 0.353	1.003 (0.984–1.021), *p* = 0.779
Depressive symptoms measured by GDS	1.714 (0.956–3.074), *p* = 0.07	1.023 (1.005–1.041), *p* = 0.014 *
Mobility measured by TT	1.439 (0.786–2.635), *p* = 0.238	0.988 (0.97–1.007), *p* = 0.213
Nutritional status measured by MNA	1.038 (0.531–2.029), *p* = 0.914	44,593 (0.999–1.042), *p* = 0.062
Frailty syndrome measured by CFS	1.464 (0.783–2.739), *p* = 0.233	1.002 (0.983–1.021), *p* = 0.869
Insomnia measured by AIS	1.364 (0.763–2.438), *p* = 0.296	1.005 (0.987–1.023), *p* = 0.567

## Discussion

### Main findings and comparison with other studies

In our study, we wanted to compare patients with and without CGs, characterise the group of CGs and establish whether they were aware of patients deficiencies which might be disclosed in the areas of CGA.

Patients with CGs achieved worse scores in ADL, IADL, AIS and CFS tests, which means that they were more prone to function worse in everyday life, suffer from insomnia and be more frail than older people who did not require the assistance of a CG. The mean age of CGs was 59 years. Most of them were women and family members, especially spouses. The majority of CGs were able to properly assess the level of senior independence in the area of basic and instrumental activities of daily living in their senior relatives. This assessment corresponded with the objective results of ADL and IADL scales. CGs also correctly identified problems with malnutrition. However, problems such as insomnia or depressive disorders were more difficult for them to recognise. The study also showed that neither age nor gender of CGs affected the correctness of their assessment of seniors in eight domains related to particular CGA tests.

Our study showing that mostly women and family members are CGs of seniors corresponds with studies carried out by Eby et al. [22] The studies conducted by Goldstein et al. indicate that CGs are able to assess the occurrence of frailty syndrome among their relatives [7]. The results of our study are comparable – not only could respondents properly assess frailty among older patients, but also their nutritional status, basic activities measured by ADL and complex living skills measured by IADL. There are also studies performed in other healthcare settings, which showed that CGs are able to assess their relatives independently in a modified CGA scale [9]. This modified CGA scale could also be considered as a potentially helpful tool in everyday general practice.

To the best of the authors’ knowledge, no studies comparing geriatric patients with and without CGs in primary care were found in Poland. So far, there have been no studies evaluating CG’s assessment of a geriatric patients in comparison with the objective CGA in Europe.

### Limitations

There are some limitations in our study that need to be taken into account. It was conducted in only one city and its surroundings, which does not allow us to generalise the results to the whole population of Poland. Furthermore, we could not exclude sampling bias. All general practices, where the study was conducted, were randomly selected but we used consecutive sampling for patient recruitment. Moreover, in our study relatively fit older patients were assessed, because bedridden seniors were excluded. CGs who participated in the study, might have cognitive, communication or literacy deficiencies which were not assessed, but which could have hindered the correct completion of the questionnaire.

### Interpretation of the study findings and implications for practice and research

We revealed that CGs are not able to precisely assess psychological problems such as depressive disorders or insomnia. A problem with identification of psychological problems by CGs may be caused by a low social awareness of depression in Poland, especially among older people [23]. It may also be caused by the fact that in Poland, depression is viewed as a kind of “embarrassing illness” and Polish people are not comfortable when talking about their emotional problems [24]. For this reason, it is worth considering and testing the effectiveness of creating CGs’ support programs similar to those conducted, for example, in the USA [25].

Different stakeholders in Poland should take into consideration that there is a lack of informational support for CGs. Doctors and nurses and also social workers and politicians should be more interested in the condition of CGs. Family therapists and support groups can help CGs with specific problems that they are struggling with, teach them how to collaborate with other family members in taking care of older people and how to cope with frustration [26].

There is a heavy work load in primary care in Poland and currently CGA is performed very rarely. In this situation, CG’s assessment can be useful and focus the doctor’s attention on a particular area of CGA where there is a need for specific clinical tests. In this way, it may be an introduction to formal methods of assessment and can enable early identification of deficiencies that are important in providing adequate care. It is clear that, e.g. for ethical reasons, patients’ evaluation should be performed by a trained and educated HPs. It would be reasonable to involve nurses in this process. However, their number in Poland is insufficient in relation to the needs, so the initial assessment of the patient’s condition by the CG and signaling senior’s deficiencies to the GP may be important. In this way, GPs are not inactive in the face of seniors’ problems, but they take into consideration the CGs’ assessment and can use these opinions in their daily work.

## Conclusions

In Krakow, Poland, CGs of seniors are mainly family members and are themselves often older people. CGs have particular problems with reliable assessment of depression and sleeplessness among relatives, but they can properly assess seniors in basic and complex living activities and nutritional status. CGs’ assessment of older patients has limitations, but overall, if the patients agrees, it can be a valuable source of information for GPs about seniors and can help diagnose important health problems.

## Supplementary Information

**Additional file 1.** A questionnaire in which the caregiver assessed the elderly patient in terms of geriatric deficits.

## Data Availability

The datasets used and analysed during the current study are not publicly available due confidentially agreement with participants, but are available from the corresponding author on reasonable request.

## Abbreviations

ADL: The Activities of Daily Living
AIS: Athens Insomnia Scale
CFS: Clinical Frailty Scale
CG: Caregiver
CGA: Comprehensive Geriatric Assessment
GDS: Geriatric Depression Scale
GP: General Practitioner
HP: Healthcare Professional
IADL: The Instrumental Activities of Daily Living
MMSE: Mini-Mental State Examination
MNA: Mini Nutritional Assessment Short Form
TT: Timed Up and Go Test

## References

[1] Śleszyński P, Wiśniewski R, Szejgiec-Kolenda B (2018). Demographic processes in Poland in the years 1946–2016 and their consequences for local development: current state and research perspectives. Geogr Pol.

[2] Pruszyński J, Putz J, Cianciara D (2016). Demographic changes in Poland in relation to changes in other countries of the European Union. Challenges for health policy. Post N Med..

[3] Sowada C, Sagan A, Kowalska-Bobko I, Badora-Musial K, Bochenek T, Domagala A (2019). Poland: health system review. Health Syst Transit.

[4] Sherman DW (2019). A Review of the complex role of family caregivers as health team members and second-order patients. Healthcare.

[5] Raymond M, Warner A, Davies N, Iliffe S, Manthorpe J, Ahmedzhai S (2014). Palliative care services for people with dementia: a synthesis of the literature reporting the views and experiences of professionals and family carers. Dementia.

[6] Pilotto A, Cella A, Pilotto A, Daragjati J, Veronese N, Musacchio C (2017). Three decades of comprehensive geriatric assessment: evidence coming from different healthcare settings and specific clinical conditions. J Am Med Dir Assoc.

[7] Goldstein J, Hubbard RE, Moorhouse P, Andrew MK (2013). Feasibility of using information derived from a care partner to develop a frailty index based on comprehensive geriatric assessment. J Frailty Aging.

[8] Goldstein J, Hubbard RE, Moorhouse P, Andrew MK, Mitnitski A, Rockwood K (2015). The validation of a care partner-derived frailty index based upon comprehensive geriatric assessment (CP-FI-CGA) in emergency medical services and geriatric ambulatory care. Age Ageing.

[9] Goldstein J, Travers A, Hubbard R, Moorhouse P, Andrew MK, Rockwood K (2014). Assessment of older adults by emergency medical services: methodology and feasibility of a care partner Comprehensive Geriatric Assessment (CP-CGA). CJEM.

[10] Applegate W, Blass J, Williams T (1990). Instruments for the functional assessment of older patients. N Engl J Med.

[11] Greenberg SA, McCabe D. Functional assessment of older adults. In: Fulmer T, Chernof B, editors. Handbook of Geriatric Assessment. Burlington, Massachusetts: Jones & Bartlett Learning; 2018. p. 231-40.

[12] Pangman VC, Sloan J, Guse L (2000). An examination of psychometric properties of the Mini-Mental Status Examination and the standardized Mini-Mental Status Examination: Implications for clinical practice. Appl Nurs Res.

[13] Yesavage JA, Brink TL, Rose TL, Lum O, Huang V, Adey MB (1983). Development and validation of a geriatric depression screening scale: A preliminary report. J Psychiatr Res.

[14] Cruz-Jimenez M (2017). Normal changes in gait and mobility problems in the elderly. Phys Med Reh Clin N.

[15] Vellas B, Villars H, Abellan G, Soto ME, Rolland Y, Guigoz Y (2006). Overview of the MNA - Its history and challenges. J Nutr Health Aging.

[16] Clegg A, Young J, Iliffe S, Rikkert MO, Rockwood K (2013). Frailty in elderly people. Lancet.

[17] Buysse DJ, Ancoli-Israel S, Edinger JD, Lichstein KL, Morin CM (2006). Recommendations for standard research assessment of insomnia. Sleep.

[18] Pachołek A, Krotos A, Drwiła D, Kalarus Z, Piotrowicz K, Gąsowski J (2020). Comprehensive geriatric assessment in primary care practices: a multi-centered, cross-sectional study in Krakow. Poland Hippokratia.

[19] Kwestionariusz ankiety dla opiekunów seniorów z Rzeszowskiego Obszaru Funkcjonalnego. https://docplayer.pl/14155589-Kwestionariusz-ankiety-dla-opiekunow-seniorow-z-rzeszowskiego-obszaru-funkcjonalnego.html. Accessed 2 Jan 2018.

[20] Wysokiński M, Fidecki W, Cybulski M, Krajewska-Kułak E (2016). Ocena sprawności funkcjonalnej pacjentów w podeszłym wieku. Opieka nad osobami starszymi. Przewodnik dla zespołu terapeutycznego.

[21] R Core Team (2020). R: A language and environment for statistical computing. R Foundation for Statistical Computing, Vienna, Austria. https://www.R-project.org/.

[22] Eby DW, Molnar LJ, Kostyniuk LP, St Louis RM, Zanier N (2017). Characteristics of informal caregivers who provide transportation assistance to older adults. PLoS ONE.

[23] Zalewska-Juzwa A, Częstochowska E (2003). Depresja w okresie okołomenopauzalnym. Pol Merkur Lek.

[24] Kużel A, Krajewska-Kułak E, Śmigielska J (2015). Percepcja depresji w wybranych grupach społecznych. Medycyna Ogólna i Nauki o Zdrowiu.

[25] Barrett G, Swanson P, Song A. Evaluation of training program for caregivers to aging adults. J Ext. 2005;43.

[26] Llanque S, Enriquez M (2012). Interventions for Hispanic caregivers of patients with dementia: A Review of the literature. Am J Alzheimers Dis Other Demen.

