# Supplementary material for: Awareness of caregivers of geriatric deficits among older people—the results of a cross-sectional study in Krakow, Poland
Source: BMC Prim Care. 2022 Jul 26;23:181. doi: 10.1186/s12875-022-01801-z (PMC9316318; doi:10.1186/s12875-022-01801-z)
Supplement: Supplementary file 1 — Additional file 1. A questionnaire in which the caregiver assessed the elderly patient in terms of geriatric deficits. [file 12875_2022_1801_MOESM1_ESM.docx]

**CAREGIVER’S ASSESSMENT OF A PATIENT**

Age: ……………...

Gender: ……………….

1. Who are you in relation to the patient?

- Spouse/Partner
- Sibling
- Child
- Daughter-in-law/Son-in-law
- Friend/Neighbour
- Other (who?) …………………………

2. Do you consider yourself to be the patient's primary caregiver?

- Yes
- No
- I can’t assess

3. Where do you and the patient live?

- In the same apartment
- In the same building, but in other apartment
- Nearby (within walking distance)
- About 10 mins by car/bus/train
- About 30 mins by car/bus/train
- About 60 mins by car/bus/train
- More than 60 mins by car/bus/train

4. Do you think that the patient requires comprehensive geriatric assessment?

- Yes
- No
- I can’t assess

5. Is the patient capable of living independently (e.g. he/she can dress himself, eat a meal or control urination and stool)?

- Yes
- No
- I can’t assess

6. Does the patient cope with the basic activities in everyday life (e.g. he/she takes medications on his own, uses the phone, does shopping)?

- Yes
- No
- I can’t assess

7. Can the patient have significant cognitive disorders (e.g. he/she has problems with memory, concentration or with finding the right words)?

- Yes
- No
- I can’t assess

8. Can the patient be depressed (e.g. he/she is sad, has lost the will to live, is unable to be happy)?

- Yes
- No
- I can’t assess

9. Is the patient fit enough to be able to get up from the chair, walk 3 meters and sit back on the chair within 13 seconds?

- Yes
- No
- I can’t assess

10. Can the patient be malnourished (e.g. he/she has recently lost weight or has no appetite)?

- Yes
- No
- I can’t assess

11. Does the patient seem fragile (e.g. he/she is weak, has problems with daily activities)?

- Yes
- No
- I can’t assess

12. Does the patient have sleep problems (e.g. he/she is unable to fall asleep, wakes up at night or is sleepy during the day)?

- Yes
- No
- I can’t assess

13. Does the patient complain about pain?

- Yes
- No
- I can’t assess

14. Has the patient’s health related quality of life decreased?

- Yes
- No
- I can’t assess

15. Do you use any form of assistance in taking care for the patient (e.g. qualified carer/Municipal Social Welfare Center/other family member, etc.)? If so, what kind of help is it?

- Yes (who is it?) ……………………….
- No
- I can’t assess

16. Would you like to use some kind of assistance in caring for the patient?

- Yes
- No
- I can’t assess

17. Has your quality of life decreased due to the care of the patient?

- Yes
- No
- I can’t assess

18. Has anyone from health care professionals talked to you about caring for a senior patient (how should you help the elderly, what problems should you look out for, how to adapt your apartment to the needs of an elderly person, etc.)?

- Yes
- No
- I can’t assess

19. Would you like to receive information on caring for senior patients (how to help the elderly, what problems should be considered, how to adapt the apartment to the needs of the elderly, etc.)?

- Yes
- No
- I can’t assess

Thank you for completing the questionnaire.
